# DUAL SENSORY LOSS AND DEPRESSIVE SYMPTOMS: FINDINGS FROM THE LONGITUDINAL AGING STUDY IN INDIA (LASI)

**DOI:** 10.1093/geroni/igac059.946

**Published:** 2022-12-20

**Authors:** Ethan Wang, Emmanuel Garcia Morales, Frank Lin, Nicholas Reed, Jennifer Deal

**Affiliations:** Cochlear Center for Hearing and Public Health, Baltimore, Maryland, United States; Cochlear Center for Hearing and Public Health, Baltimore, Maryland, United States; Johns Hopkins Cochlear Center for Hearing and Public Health, Baltimore, Maryland, United States; Johns Hopkins Bloomberg School of Public Health, Baltimore, Maryland, United States; Johns Hopkins University, Baltimore, Maryland, United States

## Abstract

Prior work suggests that vision and hearing loss may be risk factors for depressive symptoms in older adults. However, few studies have focused on the relationship of both vision and hearing loss (dual sensory loss, DSL) and depressive symptoms. Using the first nationally representative population study from LASI in India conducted from 2017-2019, Poisson models tested the association of DSL with depressive symptoms. Self-reported vision and hearing loss were associated with increased depressive symptoms (PR 1.29; 95%CI, 1.25-1.33 and 1.15; 95%CI, 1.09-1.22) after adjusting for age, sex, education, economic status, marital status, urbanicity, region, diabetes, heart disease, hypertension, stroke, and smoking. DSL had a 46% higher risk (95% CI, 1.37-1.56) of depressive symptoms. Poor vision and/or hearing loss may be a risk factor for depressive symptoms in adults in India aged ≥45 years. The risk of depressive symptoms in those with sensory loss should be investigated in further studies.

